# Intersectionality of early developmental risks and resilience after preterm birth

**DOI:** 10.1186/s40348-026-00242-3

**Published:** 2026-06-01

**Authors:** Julia Jäkel, Janika Ludwig, Britta Hüning, Ursula Felderhoff-Müser, Christoph Härtel

**Affiliations:** 1 Klinik für Kinderheilkunde I, University Hospital Essen, University Duisburg-Essen, Essen, Germany; 2 Department of Psychology, HMU Health and Medical University Düsseldorf, Düsseldorf, Germany; 3 Department of Psychology, University of Warwick, Coventry, UK; 4 Department of Pediatrics, University of Würzburg, Würzburg, Germany

## Abstract

The period from conception through preschool age represents a critical developmental window for both microbiome and brain. During this time, several modifiable factors may influence development via the gut-brain axis, including the mode of delivery, exposure to antibiotics, maternal nutrition, breastfeeding, and sensitive, emotionally responsive caregiving. Infants born preterm (<37 weeks gestation) face numerous challenges that can perturb their developing gut microbiome as well as neurobehavioral trajectories. Biological and medical risks are exacerbated by stressful social context conditions. Understanding the complex mechanisms shaping the gut-brain axis and identifying modifiable protective factors is key to help define specific target groups and critical windows for individualized prevention of adverse outcomes after preterm birth. Today, a large knowledge gap exists on (a) how gut, brain, and behavioral development interact over time, and (b) which social and modifiable factors are key drivers of this interaction that could be harnessed for treatment and intervention. Translational research on the gut-brain axis after preterm birth is critically needed. Future studies should consider intentional sampling for variation in social factors such as level of education and immigrant background to identify populations that are susceptible to microbiome modifications and provide more evidence of how interventions might optimize long-term outcomes.

Infants born preterm (< 37 weeks gestation) make up the largest paediatric patient group in Europe today (> 8% of the total newborn population) [1]. Despite improvements in perinatal care and increased survival of infants born very preterm (VP, < 32 weeks, 1–2% of all infants), their long-term neurobehavioral outcomes and quality of life have not improved over the last decades [2, 3]. Preterm birth is associated with a cluster of acute complications such as infection, inflammation, necrotizing enterocolitis (NEC), acute and chronic lung disease (respiratory distress syndrome (RDS), bronchopulmonary dysplasia (BPD)) and intracerebral hemorrhage (ICH), which cascade into 2–6 fold increased risks for chronic health problems and impaired neurobehavioral development [4]. Gut dysbiosis may remarkably contribute to short- and long-term adverse outcomes, but very little is known about this yet.

Gut dysbiosis states in preterm infants largely differ from healthy eubiosis patterns in infants born vaginally at term, fully breastfed, and not exposed to antibiotics. Key features include reduced alpha-diversity, a scarcity of anaerobic bacteria such as *Bifidobacteria*, and functional changes such as diminished capacity for metabolizing human milk oligosaccharides. While the microbiomes of many preterm infants recover during the first weeks of life and realign towards eubiosis, the specific factors that drive this positive shift in some individuals but not others remain unknown. The brain is particularly susceptible to both positive and negative environmental influences during the critical time period from preterm birth to the first two years of life, and unresolved gut dysbiosis could be a crucial determinant for long-term neurobehavioral impairment [5, 6]. Both the multifactorial causes of preterm birth (e.g., infection, intrauterine growth restriction), in-utero exposures (e.g., maternal antibiotic and probiotic use, dietary intake, stress), and postnatal invasive treatments are main risk factors for short-term complications originating in the gut such as Gram-negative bloodstream infection (BSI) or NEC. However, studies on the long-term implications of a healthy gut microbiome for infant neurobehavioral development have only recently emerged. Brain and gut microbiome develop rapidly in parallel during the first years of life and microbiome composition has been linked with typical development of cognition, behavior, and brain structure in childhood. Several mechanisms of the crosstalk between gut and brain have been postulated by animal models including (i) gut bacterial production of neuroactive metabolites and neurotransmitters, ii) bidirectional communication via neural pathways between the enteric and central nervous system, iii) hormonal production in the gut and impact on the stress axis, and iv) interaction between gut immune cells and integrity of neural networks. Studies with human adults have documented a major modulatory influence of diet on the brain-gut-microbiome relationship with important implications for neurobehavioral health. For instance, a systematic review of 15 studies using various methods including 16 S rRNA sequencing and metagenomic analysis showed that depression was associated with reduced microbial diversity and high levels of Firmicutes, while anxiety was associated with low levels of short-chain fatty acid-producing bacteria and high levels of Proteobacteria. Importantly, probiotics and dietary changes were as effective as pharmacological treatment leading to symptom alleviation in many patients [7]. However, vast variations in bacteria-brain-behavior relationships have been reported and most of the variance in the human microbiome remains unexplained. This is why experts now call for longitudinal studies that pay close attention to demographic sample characteristics to harness the great potential offered by understanding underlying mechanisms and different trajectories [8].

In fact, theories and conceptualizations about the different factors explaining long-term risk after preterm birth differ largely across disciplines. In the biomedical sciences, for example, biological and medical factors unsurprisingly represent the major focus of interest while contributions of the social environment and its interplay with biological risks and medical treatment are often underestimated. We argue that a multidisciplinary biopsychosocial approach is key to answering current open questions about the mechanisms that shape microbiome development early in life and its long-term consequences. For instance, using a person-centered cluster analysis approach, we recently documented that different risk profiles are independently associated with neurodevelopment in preterm born infants, and that social risks such as low education and language barriers are as strong in effect size as severe maternal and infant neonatal medical complications (Middendorf et al., under review). **Accordingly, we propose that social factors play a critical role for microbiome development early in life and its long-term consequences**: Maternal microbiomes differ remarkably across populations and geographic regions, but the vast majority of human microbiome data are from individuals living in WEIRD (i.e., Western, educated, industrialized, rich, democratic) countries [9]. The striking under-representation of minority groups such as immigrants in microbiome studies is a missed opportunity, because possible associations of social factors such as immigrant status or maternal education with the extent of gut dysbiosis among infants born preterm is unknown. Emerging evidence suggests that the intersectionality of social determinants of health with the microbiome shapes immigrant health outcomes [10]. This is not surprising, since international migration often results in major changes in living environments and lifestyles, including diet, while dietary and lifestyle practices emerge as the most influential factors for shaping gut microbiomes [11]. Thus, **the microbiome represents a possible mechanism linking variations and changes in social factors to health conditions**. In fact, microbiome composition has been found to vary according to country of birth, age at migration, time since immigration, and country of resettlement [7], demonstrating how the microbiome adapts to changes in social environments and lifestyle over time [11].

Human migration is at an all-time high, with 281 million people on the move worldwide [12]. Europe hosts over 87 million people who were not born in the country they reside in [12]. In Germany, for instance, almost one in two children (> 40%) live in families with at least one immigrant parent [13]. Immigrant children are growing up with a high risk for educational and mental health problems [14]. However, studies are confounded by intersectionality of inequalities such as parents’ low education, unemployment, and language barriers. The concept of intersectionality originated in Crenshaw’s critical assessments of anti-discrimination laws in the USA [15]. It here refers to the understanding that everyone lives with their own unique identities, including both visible and nonvisible identity markers that can trigger different types of experiences of discrimination, including for example gender, sexual orientation, skin colour, language skills, mental ability, and education [16]. Inequalities can be exacerbated by language difficulties that pose barriers to immigrant families’ use and received quality of healthcare and education. According to a report of the German Centre for Integration and Migration Research, more than 1/3 of ethnic minority women experienced discrimination in healthcare [17]. This has severe consequences, in particular for the youngest and most vulnerable groups, such as immigrant children born preterm who have chronic conditions and complex needs. The reasons for biases and barriers in healthcare are manifold and may include misperceptions, misunderstandings, prejudices, and unconscious biases, but also day to day demands for providers that conflict with tailored, culturally-sensitive care. At the same time, culturally appropriate and sensitive paediatric care can facilitate service utilization, reduce perceived stigma, and foster resilience [18]. 

We would like to provide a pioneering example of one of the mechanisms proposed here: In a recent study with the multi-center population-based study German Neonatal Network (GNN) cohort, we analyzed whether mothers’ immigrant status and language barriers were associated with perinatal health and short-term neonatal outcomes in 3606 infants born preterm < 32 weeks of gestation. Mixed-effects models showed that language barriers, operationalized as linguistic distance from mothers’ first languages to German, were independently associated with diagnoses of preeclampsia, while VP infants of foreign-born mothers had higher odds for amniotic infection syndrome than infants of German mothers, but there were no other associations with medical complications [19]. We have also documented that a higher linguistic distance is associated with higher neurobehavioral difficulties among children born VP at age 5–6 years [20], suggesting mediating effects of other variables being at play. Linguistic distance mainly serves as a proxy variable for systemic language barriers individuals may encounter in their daily lives. While it is also correlated with migration background and socioeconomic status, we have been able to disentangle these three factors in our previous studies to show the specific contributions of family-based language barriers to infant health. Indeed, GNN data support our hypothesis that the microbiome may be part of the underlying mechanisms. We propose that language barriers are associated with high levels of maternal stress exposures, for instance through experienced discrimination and lack of social contacts, which decreases microbiome diversity and limits microbiome establishment at the mother-infant interface [21]. In line with this, misunderstandings in communication with healthcare personnel may for instance result in inadequate application of recommendations regarding nutrition, medication, and care for preterm infants as well as reduced access to kangaroo mother care (KMC), all of which have known effects on the gut-brain axis [22]. We demonstrated an association of neonatal diseases originated in the gut, i.e., BSI with Gram-negative *enterobacterales* and NEC, with adverse long-term neurodevelopment [23]. Additionally, BSI or NEC during the neonatal period correlated with intelligence scores (IQ), behavioral difficulties including hyperactivity symptoms, and severe motor deficits (i.e., cerebral palsy, CP) at age 5–6 years. **These findings suggest that understanding the interplay of social factors, microbiome composition, and brain health during critical developmental windows will help identify effective neuroprotective strategies for infants born preterm**.

Apart from direct links with the microbiome and neurobehavioral outcomes, social factors are closely associated with mothers’ lifestyle choices such as smoking, breastfeeding, and diet. Bronfenbrenner’s bioecological systems theory emphasizes that development is shaped by interactive associations between an individual, their close social relationships, and their embedding in broader societal structures such as healthcare and education over time [24]. Accordingly, different types of risks and protective factors do not only intersect in multidimensional ways in different populations, but they also dynamically affect each other over time. For instance, considering potential protective factors, exposure to the maternal breastmilk microbiome is associated with a significant reduction in NEC among infants born preterm [25], while KMC can support both preterm infants’ [26, 27] and their parents’ health [28]. Not all social factors are modifiable, but there is an urgent need to better understand the role they play to design both individualized and systemic approaches towards more effective treatment and care. 

It is critically important to identify modifiable postnatal factors that can move the needle in optimizing long-term development to foster resilience. One such social factor that is known to be particularly protective for the long-term development of infants born preterm and modifiable is sensitive and emotionally responsive parenting [29]. Interestingly, emotional connection between mother and infant may be positively associated with gut microbiome composition, e.g., via visceral oxytocin pathways [30]. Sensitive parenting helps buffer against the negative effect of low gestation on later child outcomes [31], i.e., higher levels of sensitivity in bi-directional behaviors offer a protective effect for the most vulnerable of infants. High caregiving sensitivity promotes better cognitive functioning, behavioral, and emotional regulation for all children and is especially protective among children born at lower gestational ages [32]. In this context, it is important to note that a recent IPD meta-analysis showed that mothers of children born VP were at particularly high risk to show low sensitivity in dyadic parenting behavior [29]. This highlights a marked gap where children who need the highest levels of sensitivity to thrive may be less likely to experience such dyadic interactions. What’s more, VP children living in socially disadvantaged contexts are at highest risk of suboptimal cognitive development [33], warranting investments in interventions tailored for parents of children born VP with multiple intersectional risks such as poverty and language barriers. At the same time, this highlights the need for systematic health equity research that also address systemic issues and policies. For instance, research in non-migrant populations has shown that by formal school entry age, parents still experience elevated levels of stress and reduced family functioning after the birth of a preterm child [34], but we do not know how this experience is affected by language barriers or experiences of discrimination. A previous study with GNN data identified cultural and language difficulties as main barriers for access to early intervention programs for children born preterm [35]. Relatedly, a qualitative study of African origin mothers in the United States revealed that infant care practices were affected by immigrant mothers’ (mis)perceptions of their host society’s cultural expectations and rules [36]. For instance, results indicated that African origin mothers valued breastfeeding but often chose to use formula because they thought it was considered superior. In addition, participants highly valued carrying their infants close to the body but used equipment such as strollers because they thought it was expected [36].

We conducted a focus group discussion with five multilingual immigrant mothers and fathers of children born VP at a large Children’s Hospital in a metropolitan region of Germany to learn more about families’ lived experiences and the social factors shaping their daily lives. Participants had migrated to Germany from Syria, Ghana, Iran, Ukraine, and Portugal, all of them were fluent in English and/or German. A set of semi-standardized questions was presented to the group at the beginning in English and German. The discussion was audio-recorded and transcribed. Group interactions and atmosphere were observed and recorded simultaneously. Results were coded according to thematic content analysis and main dimensions then triangulated. Participants identified language barriers as the main process factor for further challenges for parents of preterm infants with immigrant background. In particular, access to health services was reported to be negatively affected by language problems and parents reported that language barriers regularly led to misunderstandings in care. Moreover, participants emphasized the need for greater responsiveness to their needs, enhanced access to information and relevant resources, as well as the facilitation of peer support networks for families facing similar intersectional challenges. Fig. 1 summarises the main themes and their connections with language barriers, ranging from implications on parents’ own lives (in blue) to their preterm children’s healthcare needs (in purple). Remarkably, immigrant parents frequently described a lack of social interactions with other adults and associated feelings of loneliness and isolation when dealing with their preterm child’s chronic healthcare needs. These are new and unexpected findings that require more investigation, for instance addressing the question to what extent physiological correlates of loneliness may be part of the mechanisms through which social conditions become biologically embedded in the gut microbiome.

**Fig. 1:**
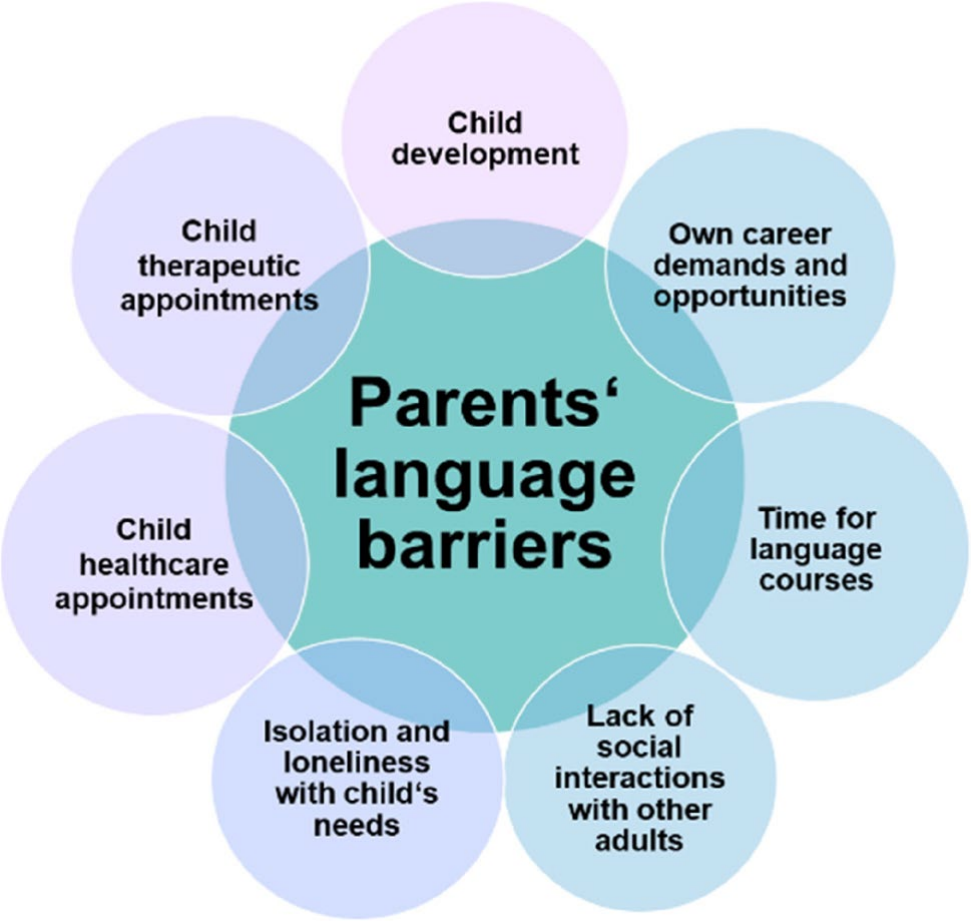
The role of language barriers for immigrant parents’ and their VP children’s lives, based on triangulated focus group results

The first few thousand days of life – the period from conception through preschool age – are recognized as foundational for neurobehavioral development. During this time, several key factors may influence development via the gut-brain axis, including social context factors, maternal nutrition, breastfeeding, and sensitive, emotionally responsive caregiving. **In the human context however, particularly after preterm birth, a large knowledge gap exists on (a) how gut, brain, and neurobehavioral development interact over time, and (b) which social and modifiable factors are key drivers of this interaction that could be harnessed for treatment and intervention**. Fig. 2 represents a hypothetical model of the potential pathways and their operationalization for study designs. Future translational research needs to include intentional sampling variation in social factors such as level of education and immigrant background to identify populations that are susceptible to alterations in their gut microbiome and provide more evidence of how interventions might optimize long-term outcomes.

**Fig. 2:**
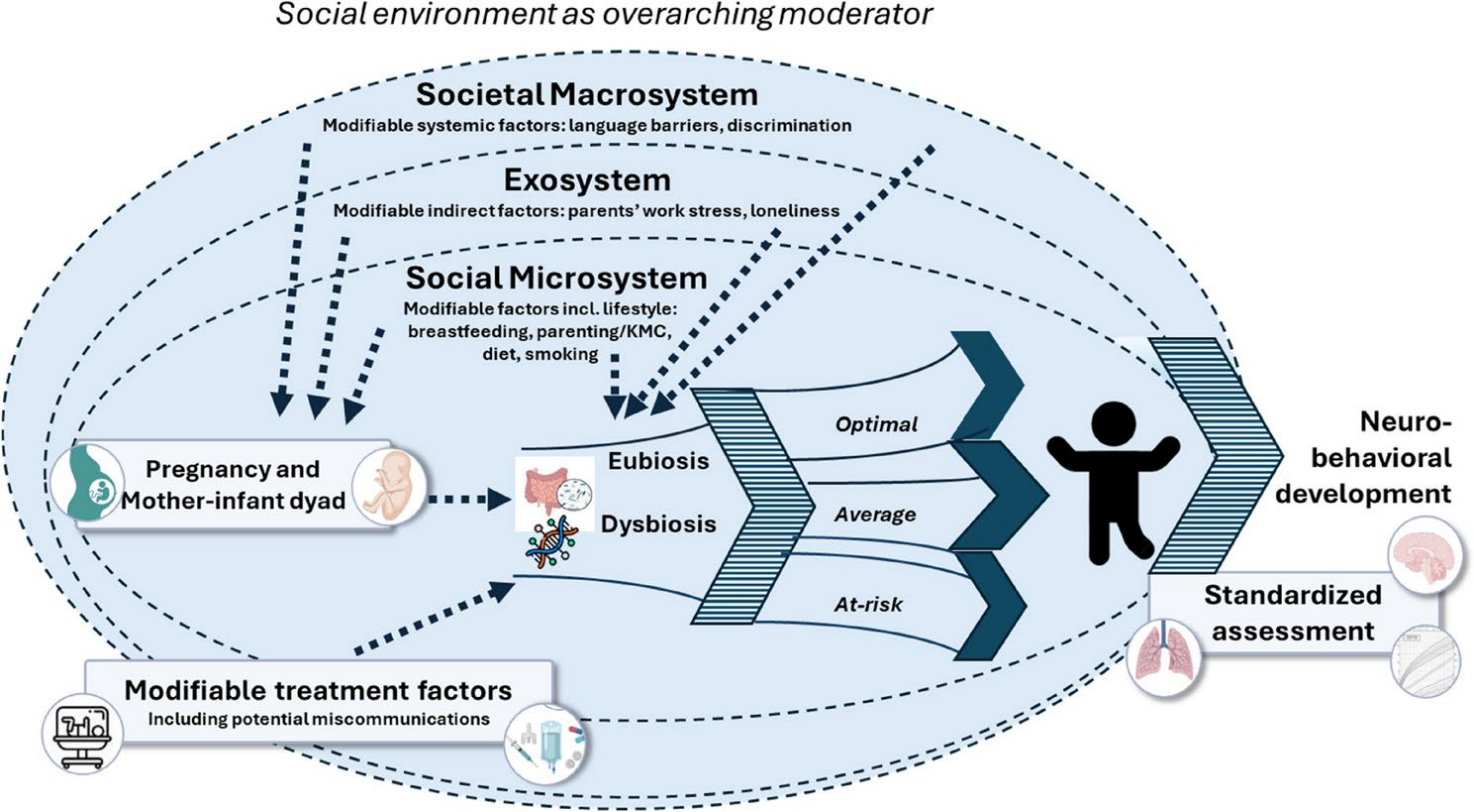
Hypothetical model of how gut, brain, social and modifiable factors are associated with neurobehavioral development over time

## Data Availability

Due to participant privacy and ethical restrictions qualitative raw data cannot be made available.
